# The Sexunzipped Trial: Young People’s Views of Participating in an Online Randomized Controlled Trial

**DOI:** 10.2196/jmir.2647

**Published:** 2013-12-12

**Authors:** Angela Nicholas, Julia V Bailey, Fiona Stevenson, Elizabeth Murray

**Affiliations:** ^1^e-Health UnitResearch Department of Primary Care and Population HealthUniversity College LondonLondonUnited Kingdom

**Keywords:** Internet, randomized controlled trials, qualitative research, outcome assessment (health care), sexual health, chlamydia trachomatis

## Abstract

**Background:**

Incidence of sexually transmitted infections (STIs) among young people in the United Kingdom is increasing. The Internet can be a suitable medium for delivery of sexual health information and sexual health promotion, given its high usage among young people, its potential for creating a sense of anonymity, and ease of access. Online randomized controlled trials (RCTs) are increasingly being used to evaluate online interventions, but while there are many advantages to online methodologies, they can be associated with a number of problems, including poor engagement with online interventions, poor trial retention, and concerns about the validity of data collected through self-report online. We conducted an online feasibility trial that tested the effects of the Sexunzipped website for sexual health compared to an information-only website. This study reports on a qualitative evaluation of the trial procedures, describing participants’ experiences and views of the Sexunzipped online trial including methods of recruitment, incentives, methods of contact, and sexual health outcome measurement.

**Objective:**

Our goal was to determine participants’ views of the acceptability and validity of the online trial methodology used in the pilot RCT of the Sexunzipped intervention.

**Methods:**

We used three qualitative data sources to assess the acceptability and validity of the online pilot RCT methodology: (1) individual interviews with 22 participants from the pilot RCT, (2) 133 emails received by the trial coordinator from trial participants, and (3) 217 free-text comments from the baseline and follow-up questionnaires. Interviews were audio-recorded and transcribed verbatim. An iterative, thematic analysis of all three data sources was conducted to identify common themes related to the acceptability and feasibility of the online trial methodology.

**Results:**

Interview participants found the trial design, including online recruitment via Facebook, online registration, email communication with the researchers, and online completion of sexual health questionnaires to be highly acceptable and preferable to traditional methods. Incentives might assist in recruiting those who would not otherwise participate. Participants generally enjoyed taking part in sexual health research online and found the questionnaire itself thought-provoking. Completing the sexual health questionnaires online encouraged honesty in responding that might not be achieved with other methods. The majority of interview participants also thought that receiving and returning a urine sample for chlamydia testing via post was acceptable.

**Conclusions:**

These findings provide strong support for the use of online research methods for sexual health research, emphasizing the importance of careful planning and execution of all trial procedures including recruitment, respondent validation, trial related communication, and methods to maximize follow-up. Our findings suggest that sexual health outcome measurement might encourage reflection on current behavior, sometimes leading to behavior change.

**Trial Registration:**

International Standard Randomized Controlled Trial Number (ISRCTN): 55651027; http://www.controlled-trials.com/isrctn/pf/55651027 (Archived by WebCite at http://www.webcitation.org/6LbkxdPKf).

## Introduction

The incidence of sexually transmitted infections (STIs) among young people in the United Kingdom is increasing, despite an overall decrease [1]. More effective interventions aimed at reducing the incidence of STIs in young people are therefore clearly needed.

The Internet is a suitable medium for the delivery of sexual health information and other sexual health promotion tools, given its high usage among young people and its potential anonymity and ease of access. Computer-based interventions for sexual health promotion can have an impact on sexual health outcomes including knowledge, safer sex, self-efficacy, intention, condom use, and partner numbers [2-4], although stronger evidence is needed to be certain of these effects and to understand how interventions may work.

Online trials are increasingly being used to evaluate online interventions [5]. Conducting trials online has a number of advantages when compared with more traditional trial methods [5,6], including the ability to recruit large numbers of participants over the Internet in a relatively short period of time [7,8] and at lower cost [5,7], recruitment of groups not usually recruited using other methods [6,9], instantaneous data collection [5,6], reduced burden on participants [10], and increased participant anonymity [8], which may be particularly important when providing sensitive information about sexual health [11].

While there are many advantages to using online methodologies for conducting randomized controlled trials (RCTs), online trials can be associated with a number of problems, including poor engagement with online interventions [12], poor trial retention [5,9,13], and concerns about the validity of data collected through online self-reporting [8]. As online trials and online data collection become increasingly common, it is important to determine the best ways of addressing these kinds of problems and to further knowledge about the best ways of conducting research online.

We conducted an online feasibility trial that tested the effects of the Sexunzipped website for sexual health compared to an information-only website. The trial was designed to test the best methods of recruitment, retention, contact with participants, and sexual health outcome measurement [14]. This study reports on a qualitative evaluation of the research procedures [15], reporting trial participants’ experiences and views of the Sexunzipped online trial.

The aim of this qualitative study was to determine the acceptability and validity of the online trial methodology used in the pilot RCT of the Sexunzipped intervention. More specifically, our purpose was to determine young people’s views on participating in an online RCT, receiving and returning a urine testing kit for genital chlamydia via post, and the importance of receiving incentives for participation. The results of this study will inform the design of a full RCT of the Sexunzipped sexual health online intervention, but also provide useful information for other researchers designing online trials.

## Methods

### Overview

The Sexunzipped intervention site is an interactive, tailored sexual health website for young people [16]. It was designed according to principles of behavior change theory [17] and was developed in collaboration with young people [18]. The website aimed to provide young people with the tools to make informed decisions about their sexual health, encouraging both safer sex behaviors and greater satisfaction with relationships and sexual choices. The site provided information under the broad categories of “relationships”, “safer sex”, and “sexual pleasure”.

### The Randomized Controlled Trial Design

The design of the RCT of the Sexunzipped website is described in Textbox 1 (see also Multimedia Appendices 1 and 2). Quantitative outcomes of this pilot trial are reported in the companion paper [14].

**Summary of the Sexunzipped online pilot RCT.**

*The study*

We conducted an online randomized RCT to test the hypothesis that the Sexunzipped theory-based, interactive online intervention would be more effective in promoting sexual health in young people than an information-only comparator website. Ethical permission for the study was granted by the University College London Ethical Committee (ref: 1023/002).

*The websites*

The primary difference between the “intervention” and the “control” website was the presence of interactive content on the intervention website. The control site presented simple factual information only, while the intervention site encouraged active engagement and self-reflection through quizzes and decision-making activities that were absent from the control site.

*Recruitment*

We invited young people aged 16-20 years living in the United Kingdom to participate in the study by placing advertisements on sexual health websites, the social networking site Facebook, on UK school and college notice boards, and by distributing flyers outside three sexual health clinics and one school for students over 16 in London, UK. We also emailed study participants to ask them to invite friends to participate.

*Online enrollment and consent*

Young people enrolled via the Sexunzipped website, which offered a £10 incentive for participation. Once they provided consent online, participants created a username and password and were directed to a baseline demographic and sexual health questionnaire.

*Participants*

In total, 2036 participants provided consent to participate in the trial, recruited from all four countries of the United Kingdom and Northern Ireland. After removal of duplicate or invalid registrations, 2006 people participated in the online trial (age range 16-21 years, median age 19; females=62.81%, 1260/2006; males=36.59%, 734/2006; transgender and “other”=0.60%, 12/2006). For a detailed discussion of the methods used for removal of duplicate and invalid registrations, see the companion paper [14].

*Baseline and follow-up questionnaires*

Demographic information including email address and postal address was collected online at baseline. Participants also completed the Sexunzipped Sexual Health Questionnaire, which measured knowledge, self-efficacy, intention, and behaviors relating to safer sex and communication, sexual and relationship problems, and satisfaction (Multimedia Appendix 1). Participants were contacted by email at 3 months and invited to click on a hyperlink to complete the follow-up sexual health questionnaire, which was identical (Multimedia Appendix 2: email wording). The overall response rate for submission of the follow-up questionnaire was 71.78% (1440/2006).

*Randomization*

After completing the baseline questionnaire, 1034 participants were randomized to the intervention and 972 to the comparator website. All were given unlimited access to their allocated website during the course of the study. Approximately three-quarters of participants (1520/2006) accessed their allocated website at least once.

*Postal chlamydia tests*

Half of the participants (n=1030) were randomized to receive by mail a urine pot to test for genital chlamydia at 3 months. Participants returned the urine sample using a pre-paid return envelope. Nonresponders received one repeat postal kit. Participants could choose to receive the test results by text, phone, or mail. The return rate for the chlamydia sample pots was 44.85% (462/1030).

*Incentives*

In a substudy to increase retention, 902 participants were randomized after recruitment but before follow-up to receive a £10 (438/902) or a £20 (464/902) incentive for completion of either the follow-up questionnaire (417/902) or completion of the follow-up questionnaire plus return of the chlamydia test (485/902). The greater incentive boosted completion rates by 6-10%.

### Qualitative Study Design

We used three qualitative data sources to assess the acceptability and validity of the online pilot RCT methodology: (1) individual interviews with 22 participants from the pilot RCT, (2) emails received by the trial coordinator from trial participants, and (3) free-text comments on the online baseline and follow-up questionnaires.

### Data Source 1: Interviews

#### Recruitment

The last question at the end of the 3-month follow-up questionnaire asked trial participants whether they would be interested in participating in an interview regarding their participation in the Sexunzipped online trial. Of the 1205 trial participants who responded to this question, 583/1205 (48.38%) said they did not want to participate in an interview, 167/1205 (13.36%) stated they would like to participate, and 455/1205 (37.76%) said they would like more information before deciding. The postal codes of those participants who stated they would like to participate or would like more information were analyzed to identify the locations of clusters of potential interview participants. Five geographical clusters were identified (described below), and those trial participants who indicated interest and who were residing in these areas were emailed to invite them to participate in an interview. With the exception of one interview (conducted by JB), interviews were conducted by researchers who had not been involved in the development of the Sexunzipped site or in the administration of the online trial (AN and CS). Participants were made aware of this.

Interviews were conducted in five locations across the United Kingdom to help achieve a maximum variation sample and to help increase transferability of findings. The five cities were chosen because there were clusters of participants in each [14] (generally related to the presence of one or more large universities) and because they represent vast differences in level of deprivation and affluence, population, ethnic mix, and geographical profile. Glasgow is the largest city in Scotland (population approximately 600,000 in 2011) [19] but also has one of the highest unemployment rates in the United Kingdom. Liverpool (population approximately 455,400 in 2011) [20] is one of the largest cities in England but has the highest level of deprivation of any English city. Bristol (population approximately 440,000 in 2012) [21] is in England’s Southwest and is England’s eighth largest city; it has a large population of 20-29 year-olds, probably owing to its large tertiary student population. Southampton (population approximately 236,900) [22] is a relatviely small English city located on the southern coast of England. Compared with other cities in the United Kingdom, unemployment in 2012 was relatively low. Manchester (population approximately 503,100) [23] is one of England’s largest cities, but rated 4th on the index of multiple deprivation in 2010. London (population approximately 8,173,900) [22] is England’s capital city and had the highest level of disposable income of any UK city in 2010.

We aimed to gain a maximum variation sample of the trial participants in terms of age, gender, allocation to the intervention or control site, and allocation to receive a chlamydia test in order to gain feedback from a diverse range of participants. As the recruitment process proceeded, we undertook more purposive sampling by specifically targeting particular groups who were underrepresented in the interviews, such as males, participants who had received the chlamydia test, and younger trial participants, until distribution of these characteristics of interview participants better reflected those of the pilot RCT sample. We continued to recruit participants for the interviews until data saturation, in other words, until there were no new themes emerging regarding participants’ experiences of the online trial methodology.

#### Interview Procedures

All participants were interviewed face to face. The interview content included questions regarding the acceptability and feasability of the online trial methodology (reported here), as well as participants’ opinions on the website itself (not reported in this paper). The latter required participants to engage with the Sexunzipped website, with the interviewer directly observing. The researchers therefore chose to conduct interviews face to face, rather than via online means such as Skype or an online chat facility.

Interviewers used a topic guide for the semistructured interviews, which reflected our research agenda and also allowed scope for participants to expand on topics and themes as they chose. Interviewers also encouraged participants to raise other issues regarding the trial that had not been prompted but they thought important to discuss. The topic guide covered participants’ experiences of being in the online trial and of receiving a postal chlamydia-testing kit.

Written consent was gained from all participants to record the interview and for the use of anonymous quotations. All interviews were audio-recorded and sent to a professional transcriber for verbatim transcription. Interviewers’ field notes were also used in conjunction with the transcripts in the interview analysis.

#### Interview Setting

The interviews were conducted in a variety of setttings including in a seminar room at a sexual health center, in university offices, in a seminar room at a community center, and in a commercially rented office. Participants were interviewed alone, apart from 2 participants (close friends) who requested to be interviewed together.

#### Analysis of Interview Data

All interview transcripts were coded and analyzed using a thematic analysis technique and using Atlas.ti software (Version 6) for data management. Three researchers coded one of the manuscripts and compared coding decisions to finalize the coding schema to be used. The rest of the analyses were undertaken by one of the researchers who had conducted a number of the interviews (AN). Transcripts were initially coded as being responses to a particular question and subsequently free-coded by theme. Thematic coding occurred iteratively, with themes emerging throughout the analysis. Once all transcripts had been coded in this way, codes were grouped and common themes identified. Emergent themes were discussed with other researchers at intervals throughout the coding process, with clear themes emerging early in the analysis process.

### Data Sources 2 and 3: Participant Emails and Questionnaire Free-Text Comments

#### Data Collection and Procedures

Throughout the course of the trial, the trial coordinator saved all emails received from participants that asked questions or provided comments about the trial. These emails were sorted into folders based on their content. Those that concerned questions or comments on trial participation or trial procedures were used as a dataset for this qualitative study. The trial coordinator was sent 133 emails from trial participants in relation to the trial methodology.

On both the baseline and follow-up RCT outcome questionnaires (filled in online), participants were provided with a space to add any free-text comments. At the end of the study, the researcher extracted these comments from the questionnaires and used these as a dataset for qualitative thematic analysis; 180 free-text comments were made on baseline questionnaires (out of 2006 submitted questionnaires) and 109 on the 3-month follow up questionnaire (out of 1440).

#### Data Analysis

AN analyzed the content of participant emails and the questionnaire free-text comments. This was done by initially identifying those emails and comments related to the trial methodology and then using an iterative, thematic analysis approach to identify common themes across the emails and the questionnaire free-text comments. This was done manually. The results of the analysis of the free-text comments and emails were considered in combination with those of the interviews. These data tended to further illustrate themes that had arisen in the interviews.

## Results

### Overview

These results represent findings from all three data sources described in the Methods section. Results are presented thematically, rather than by data type, but the data type is specified in each section.

### Interview Participants

Interviews were conducted with 22 participants aged 16-20 years who had participated in the online pilot RCT of the Sexunzipped website. Demographic characteristics of these participants are outlined in Table 1. The median age of interview participants at the time of trial registration was 19 years (range 16-20). The median age and distribution of gender reflect that of the pilot RCT sample (see Textbox 1 and the companion paper [14] for further details of the demographics of the pilot RCT participants). More than two-thirds of participants (77%, 17/22) were white British, also similar to the pilot RCT sample (84.20%, 1679/1994). Almost all participants were either currently participating in, or had just completed, either high school or a university degree. They were predominantly undergraduate students. An equal number of participants were interviewed from the intervention and control conditions and similar numbers of participants were interviewed from the chlamydia-test and no chlamydia-test groups.

**Table 1:** Interview participant demographic characteristics (N=22).

Characteristics		n (%)
**Gender**		
	Female	15 (68)
	Male	7 (32)
**Ethnicity**		
	White British	17 (77)
	Black Caribbean	1 (5)
	Mixed cultural background	1 (5)
	South American	1 (5)
	White other	1 (5)
	White Irish	1 (5)
**Location**		
	London	10 (45)
	Liverpool	4 (18)
	Bristol	3 (14)
	Manchester	2 (9)
	Southampton	2 (9)
	Glasgow	1 (5)
**Website allocation**		
	Intervention	11 (50)
	Control	11 (50)
**Chlamydia test**		
	Sent postal sample pot	10 (45)
	No sample pot	12 (55)

### Participating in Online Research

#### Reasons for Participating

Almost all of the interview participants stated that they were recruited to the RCT through an advertisement on Facebook (overall, 84.0%, 1685/2006 of the pilot RCT participants were recruited via Facebook) [14]. The most common reasons for wanting to participate were to help the researchers because they understood it is difficult to find participants or because they liked to help other people in general, and to gain the voucher offered as an incentive. Some participants who felt they were from “minority” sexualities (identifying themselves as gay, bisexual, polyamorous, and/or as having a transgender sexual partner) stated they wanted to represent their sexualities in the research:

As a gay man…I feel it’s important to get a fair representation, so I felt like my opinion was important.

Participant 1278, male, 20 years

A number of participants expressed an interest in sexual health as their primary motivator for participation. Other participants described participating simply because they like to take part in studies: “I just generally say ‘yes’ to these things” [Participant 427, female, 18 years]. Some interviewees stated that they participated because they were psychology or sociology students, encouraged to participate in research to learn about research methods and processes, or because they thought it would be fun or interesting to “take part in a sex survey” [Participant 494, male, 16 years].

When asked directly if they would have participated without the offer of incentives, the majority of participants said that they would have because they had other motives for participation. However, those who participated in the trial purely for the incentive seemed to differ from those who participated for other reasons in that they tended to be studying topics unrelated to health or social welfare and they also tended not to have participated in much research in the past.

#### Participants’ Understanding of the Purpose of the Research

Despite having indicated they had read the information sheet that provided a clear overview of the trial purpose and procedures and having provided written consent to participate in the trial, the interview participants most commonly thought (incorrectly) that the purpose of the research was to gain data on the sexual health of young people, to test the sexual health knowledge of young people, or to measure attitudes to sex. Only one person thought, correctly, that the research was conducted to determine whether the Sexunzipped website would promote sexual health behavior change, and one further person was partially correct in thinking that the study would help to create a better website.

#### Who Was Running the Research and Is That Important?

When asked whether they knew who was running the Sexunzipped research project, about half of the interview participants correctly said it was University College London (UCL). An almost equal number, however, did not know who was responsible; 2 participants knew it was a university, but were not sure which one.

About a third of interview participants said that it did not matter to them who was running the research, while another third said that they knew UCL was a well-regarded university and that was important to their decision to participate. A number of participants thought it was important that it was a university running the research, but it did not matter which one. Two participants said it was only important that the research was not being run by a commercial company:

I think if it had been a corporate company doing it, I think I would still have done it, but I kind of would have approached it with a different attitude, I guess, if I thought they were trying to sell something.

Participant 595, female, 19

#### Experience of Completing the Research Online

No participants reported negative experiences of participating in the research. All participants reported either a positive experience or a “fine” or neutral experience. All participants said that they would participate again.

No interview participant reported any negative attitudes to contact via email. On the contrary, they frequently expressed a preference for email over mail or phone, citing the convenience and time-saving aspects of email, as well as the feeling of anonymity. Participants also raised no objections to the specific content of the emails.

In regard to the questionnaires being presented online, interview participants again expressed their appreciation of the electronic format. Specific comments articulated the ease of completing the questionnaires at any time the participants had an Internet connection (on a laptop, university library, at home) and of being able to press a button and have their responses submitted without any further effort. Participants were clearly comfortable with online communication and data collection.

#### Attitudes Toward the Sexual Health Questionnaire

Despite the considerable length of the online questionnaires and the inclusion of detailed personal questions about participants’ sexuality and sexual behaviors, both trial and interview participants were generally positive about the questionnaires. Comments provided by the pilot RCT participants directly on the sexual health questionnaires suggest that they were highly engaged with the questionnaires. About a third of comments provided on the baseline questionnaire and more than two-thirds on the follow-up questionnaire expressed positive views about the questionnaire. The most common positive comments related to participants finding the content and range of the questions interesting, that the questions challenged participants’ thinking about sex and sexual health, and the inclusivity of the response options. Positive comments about the online format being easy to use were also relatively common.

Less positive, though constructive, comments commonly made suggestions for additional response options to questions, particularly “middle of the road” options such as “maybe” or “not sure” and for additional clarification of questions or terms used within questions, such as what actually constitutes “sex”.

A relatively large number of comments provided additional details to the multiple-choice responses in the questionnaire, suggesting a desire from participants for their answers to be understood in context. For example, explaining a “Not applicable” response by stating “I have only had sex with my recent husband”. Participants also seemed concerned that researchers not judge them negatively, frequently providing comments in defense of their responses: “I feel like I may come off as someone who doesn’t care about STIs and such. This isn’t true. Yes I have multiple partners, but every 6 weeks we all go to the sexual health clinic together”.

#### Being Asked About Sex and Relationships

Interview participants were asked if they minded being asked about sex and relationships on the Sexunzipped online questionnaire. Only one person expressed any concern about this, and her concerns related to questions regarding confidence to discuss sex in relationships. While some interview participants simply thought the questions were “no big deal”, some found the questions to be really “fun and interesting” and liked the directness of the questions. A number of interview participants said that participation in a sexual health study implied being asked these sorts of questions:

When you signed up, you…realized what you were going to be asked, so there was nothing shocking.

Participant 985, male, 18 years

Others said that, while they might have felt confronted or somewhat shocked by some of the questions, they appreciated those questions:

If I was shocked by anything I was…glad to see it because…we should talk about everything, and not be scared to talk about these things.

Participant 1278, male, 20 years

#### Honesty in Responding and the Importance of the Questionnaire Responses

All interview participants said they responded honestly to the questionnaire, and all but one participant stated that they believed that the responses they provided on the questionnaires were important to the research. A number of interview participants referred to the sense of anonymity afforded them by completing the questionnaire online and stated that they would not have felt so comfortable in responding honestly if they had needed to hand their completed questionnaires to a person, or to complete their responses with others present:

…because it was online and no one was asking you anything to your face, it was sort of easy just to answer as honestly as you could. So, I think that was good…I think if someone had been asking me that face to face, I don’t think I would have been as honest!

Participant 1072, female, 19 years

Once again, those interview participants who saw their sexuality as somewhat “unusual” or in the minority expressed that being honest allowed them to be represented in the research. Some interview participants simply thought that lying would be pointless or would require more effort than just telling the truth.

Several interview participants referred to aspects of the questions themselves that facilitated honest answers. For example, because the questions always provided a response option that allowed them to provide an honest response (ie, not forced into an “approximate” response through the forced-choice options). Furthermore, some interview participants also referred to the wording of the questions in facilitating honest responding:

even things like…‘how many times in the last 3 months have you had sex without a condom?’…The amount of times we are told in school…that that is strictly forbidden… the fact that they ask it in such a comfortable and normal way…it’s just easier to be honest that way.

Participant 1519, male, 19 years

#### Did Completing the Questionnaire Change Sexual Health Behaviors?

Interview participants were asked whether completing the sexual well-being questionnaire had made them think differently about their sexual health and whether they had changed any behavior relating to their sexual health as a result. The majority of interview participants stated that in order to answer the questionnaire honestly, they had reflected on their own behavior and that some particular questions had made them think very carefully about some aspects of their sexual well-being. Comments made by the pilot RCT participants on the online questionnaires also illustrated that the questions had changed their thinking about sexual health, including comments such as “challenged my thinking”, “made me think more”, and “Made me think more about my sex life and that I need to take more care and be protected more often”.

A number of the interview participants had given particular thought as to whether they were comfortable talking to their partner about sex. Some reported thinking more about contraception and their attitudes towards different types of sexual practices (eg, oral sex, anal sex, masturbation, use of sex toys). Other interview participants said that they had given particular thought to control in relationships, past relationships in general, sexual health services available, sex and alcohol, sexuality, pressure to have sex and regret after sex:

I’d had, like, an experience in the past where I’d kind of felt a bit more pressured into it…it (the questionnaire) did make me think… should I be more aware of that in the future and maybe I can do something to prevent that feeling or that situation

Participant 1278, male, 20 years

When asked if they had acted differently as a result of completing the questionnaire, about two-thirds of interview participants said they had not. Most frequently, this was because they felt that no changes needed to be made, either because they were already “careful”, were in a long-term monogamous relationship, or not currently sexually active. Only one participant felt that he needed to make changes but had not. Those interview participants who said they had changed their behavior consequent to completing the questionnaire (about a third) had changed behavior relating to using contraception, being more careful about using contraception for sex while drinking alcohol, standing up to pressure to have sex, not having “casual sex” they might regret, or being screened for STIs.

### Postal Tests for Genital Chlamydia

Approximately half of the overall pilot RCT participants received a test in the post for genital chlamydia. All interview participants were asked how they felt about having to provide their address as part of the research and whether they understood why their address was needed. Those who received the test were asked how they felt about it, and those who had not received the test were asked if they would have minded receiving the test via the post.

Only one interview participant stated that she did not like having to provide her address. This was because she was concerned her parents would find out about her participation in the study. While the majority of interview participants did not mind providing their address, a number said that was because they lived with friends or at a college, but they would be more concerned if they lived with their parents. This concern was also exemplified in several emails sent to the research coordinator from pilot RCT participants asking if post relating to the Sexunzipped study (the urine test package, voucher, or postal questionnaires) would display a Sexunzipped logo on the package as the trial participants were concerned that their parents would learn of their participation in the study.

A number of interview participants also specified that they did not mind providing their address because they trusted UCL as a legitimate organization or because they understood the need for the address:

But I did understand that it might actually have something to do with the research, so I didn’t really mind… I mean, I’ve given my postal address for worse things, like for adverts and things, when I’d just learned about the Internet… And also you guys were a legitimate research body. So I wasn’t scared, you know, oh, maybe they’re going to sell my postal address, or they will try and steal my identity.

Participant 489, male, 20 years

Of the interview participants asked to state why they thought the research team needed their address, approximately half believed it was to receive the postal chlamydia test, a few thought it was to receive the shopping vouchers received as incentives, two thought it would be used to collect data about participants’ location, and the remainder were uncertain why they needed to provide an address (but provided it anyway).

More than two-thirds of interview participants either did not mind or would not have minded receiving the chlamydia test in the post. Many of the interview participants had completed genital chlamydia urine tests prior to participating in the study and found the testing “pretty standard”. Participants had sometimes received testing kits via post from the National Health Service or had picked them up from family doctors, sexual health clinics or nightclub bathrooms, or received kits at university:

I’ve received them loads of times from the NHS

Participant 1072, female, 19 years

I’d have been fine doing it, because we did them at...we had the people come round our uni quite recently anyway to do chlamydia tests and we got like a free T-shirt if we did and stuff like that.

Participant 427, female, 19 years

A number of interview participants obtained chlamydia tests regularly anyway: “I’d just cross ‘Chlamydia test’ off my checklist” [Participant 1101, female, 18 years]. Two participants thought that receiving a test was not appropriate: “It’s kind of intrusive…[you] should go to your local clinic” [Participant 734, female, 19 years].

Several emails sent to the research coordinator from the pilot RCT participants specified concerns or questions regarding return of the urine sample. Some trial participants had recently completed a chlamydia test and asked if they therefore needed to be tested again. One participant had never had sex and wanted to know if he should return the sample anyway. Another participant wanted further explanation as to what the urine sample would be used for before making the decision about whether to return it.

### Incentives for Participation

Of the 22 participants interviewed, most had received one £10 voucher, one had received the £20 voucher, and the remainder had received the £10 total in two £5 increments, with the exception of one participant who had not yet received any vouchers and one who could not recall the amount he had received.

The majority of participants thought the £10 they received was an adequate incentive, while the participant who received £20 thought that amount was too much for what she had been required to do as part of the research. While participants were glad to receive such an incentive, the majority of them also stated that they would have participated anyway with no incentive.

## Discussion

### Principal Findings

This evaluation of young people’s views of the methodology used in an online pilot RCT demonstrates that the online methodology used was highly acceptable to this group and is in fact preferred to “traditional” face-to-face or postal methods for sexual health research. Recruitment online via Facebook proved to be effective for the age group 18-20 years, and this recruitment method was highly acceptable to participants. However, we could not recruit young people aged 16-17 years via Facebook since Facebook prohibits advertisements with reference to sex or sexual health to those under 18 years old.

Participants’ main motivations for participation were a desire to aid the research, to gain the incentive, and an interest in the area of sexual health. Despite the incentive being identified as a common motivator for participation, the majority of interviewees stated that they would have participated without it. This is consistent with previous research that suggests that altruistic motivations for participation in research are common, such as wanting to contribute to scientific knowledge, particularly if the risk and burden of participation is low [24]. Interview participants said that having to complete the sexual health questionnaires was not particularly burdensome, and on the contrary, could be fun, interesting, and thought provoking.

It is important to note that almost all of the interview participants were university students with an interest in research and studying health- or welfare-related degrees. Incentives may have been important for attracting participants without specific interests in the research process: interviewees who rated the incentives as most important to participation were studying in a non–health-related field and had not participated in prior research.

Contact by email was highly acceptable, and also postal contact, as long as the sexual health content was not obvious to anyone other than the recipient. We did not collect telephone numbers, but this may have boosted retention beyond the maximal 77.2%, 166/215 (achieved with a £20 incentive) [14]. Bull et al maximized trial retention by using a series of incentives and by contacting participants in several different ways (via email, post, and telephone) [9].

The young people interviewed thought it important that studies of the sexual health of young people are conducted and wanted to help by providing valid data. Many comments on the sexual health questionnaires also expressed positive reactions to the broad range of questions asked and indicated strong engagement with the questionnaire, suggesting that they were keen to provide accurate responses to the questions.

The information participants were asked to provide was of a highly sensitive nature (eg, types of sexual activity, number of sexual partners, history of sexually transmitted infections), but interview participants were comfortable with providing this information anonymously online. All participants said they provided completely honest answers to the questions, but they might not have done so if they had to hand in a written questionnaire or if asked for that information by a person (face to face or by phone). This finding is consistent with that of Copas et al [11] who concluded that use of Computer Assisted Self Interview technology compared with pen and paper completion improved data accuracy for a survey of sexual attitudes and lifestyle in a British population. This suggests that information collected online is likely to be at least as valid as information collected offline.

A majority of interview participants said they were more willing to participate in the research because it was university-run. For many participants, their confidence was enhanced by the knowledge that the research was being conducted by a reputable university and not by a commercial company. Beyond this, the details of the research were not important to them, with few participants understanding the purpose of the study and only half being able to name the university responsible, despite these details being clearly provided in all recruitment materials.

Participants expressed a preference for online registration processes, communications with researchers via email, and completion of the questionnaires online, seeing these as convenient in terms of being able to participate at a time and place of their choosing and in affording them a maximum sense of anonymity. The online environment was also valued by participants in an online trial of an alcohol harm reduction website (Down Your Drink) [10].

All participants had reflected on their own sexual health behavior to complete the questionnaires, and for about a third of participants interviewed, this had resulted in their changing their sexual health behavior. This illustrates the high likelihood of reactivity to assessment, which is essential to consider when baseline data are collected prior to an intervention [25]. It is well known that assessment of alcohol consumption alone can significantly reduce alcohol consumption [26], and further work is needed to determine the likely level of effect of reactivity of assessment in a sexual health context. Collecting only a minimal number of sexual health outcomes at baseline (to allow the examination of baseline differences between groups), with full outcome measurement at follow-up, would help to minimize measurement effects.

The majority of our interview participants thought that receiving and returning a urine sample for chlamydia testing was acceptable. However, the maximal return rate of the tests in the online trial was relatively low (47.4%, 118/249) [14]. Interview responses and email queries suggest that, in a number of cases, participants might not have returned their tests because they did not need the result, either because they were not sexually active or because they had a recent test. The companion paper [14] provides further data and discussion on this issue. A small number of interview participants thought that receiving the chlamydia test by post was too intrusive. In such cases, it might be useful to offer an alternative method of testing, such as attending a sexual health clinic.

### Limitations

The 22 interview participants, while being a diverse sample in terms of sexual preference (including gay, straight, bisexual, polyamorous, and with transgender sexual partners), allocation to intervention or comparator, geographical location, and gender, were not representative of participants in the Sexunzipped trial. They were in the upper end of the age range of the target audience, were mostly undertaking university studies, and the majority either had a specific interest in sexual health and/or in being part of research studies. This group therefore represents participants who are highly educated and motivated to participate in sexual health research, and their opinions may therefore differ from those from the broader RCT pilot sample. Furthermore, by recruiting interview participants via the follow-up questionnaire, we could not include participants who had registered for the study but dropped out. We were therefore unable to determine if aspects of the methodology were unacceptable to participants who dropped out of the study. Greater attempts to follow up with those who drop out of online studies would provide more complete information regarding aspects of these studies that may lead to attrition from online research.

### Future Directions

Recommendations for the conduct of online randomized controlled trials and online sexual health research can be found in Multimedia Appendix 3. These recommendations were derived from this qualitative evaluation and from the linked quantitative evaluation of the Sexunzipped trial procedures reported in the companion paper [14].

### Conclusions

This study contributes an increased understanding of common problems and concerns related to the conduct of online sexual health research through analysis of the views of young people who participated in the Sexunzipped trial (expressed in in-depth interviews, free-text comments on an online questionnaire, and in trial-related emails).

The online recruitment of young people through Facebook was highly acceptable to the interview participants. Similarly, online trial methodology such as online registration, email reminders and communication with the researchers via email, and completion of questionnaires online were preferred above more traditional methods. The findings of this study also suggest that online data collection for sensitive information such as sexual health data may assist in gaining valid and complete data in comparison to offline methods. Our findings suggest that sexual health outcome measurement may in itself prompt reflection or behavior change, so it is important to consider potential measurement reactivity in the design of an RCT. The provision of incentives for participation in sexual health research online may help to access harder-to-reach groups who may not normally participate.

Notwithstanding the limitations of self-selection into this study, these findings provide support for the use of online research methods for sexual health research, emphasizing the importance of careful planning and execution of all trial procedures including recruitment, respondent validation, trial-related communication, and methods to maximize follow-up.

## Multimedia Appendix 1

Sexunzipped sexual health questionnaire.

## Multimedia Appendix 2

Sexunzipped online trial - example email content.

## Multimedia Appendix 3

Recommendations for the conduct of online randomized controlled trials and online sexual health research.

## Abbreviations

RCT: randomized controlled trial
STI: sexually transmitted infection
UCL: University College London
