# Supplementary material for: The Sexunzipped Trial: Young People’s Views of Participating in an Online Randomized Controlled Trial
Source: J Med Internet Res. 2013 Dec 12;15(12):e276. doi: 10.2196/jmir.2647 (PMC3868966; doi:10.2196/jmir.2647)
Supplement: Supplementary file 1 [file jmir_v15i12e276_app1.pdf]

The Sexunzipped sexual health questionnaire

User account

To receive your **£10 shopping voucher**, we need your name and address (only one voucher per household).

Please also enter your email address.

Your details will be kept secure, in accordance with the Data Protection Act 1998.

Account information

E-mail address:

Personal details

First name:

Last name:

Address Line 1:

Address Line 2:

City:

Post code:

**Question**	
How did you hear about this research?<br><br>Facebook advert<br>Through a friend or relative<br>By email<br>Online (e.g. blog, twitter)<br>From school or college<br>Leaflet or poster<br>Other	
Are you…….<br><br>❍ Male<br>❍ Female<br>❍ Female to male transgender<br>❍ Male to female transgender<br>❍ Other (please state) ……………………………	
Have you felt sexually attracted …..<br><br>❍ Only to females, never to males<br>❍ More often to females, and at least once to a male<br>❍ About equally often to females and to males<br>❍ More often to males, and at least once to a female<br>❍ Only to males, and never to females<br>❍ I have never felt sexually attracted to anyone	
Which of these is true for you at the moment?<br><br>❍ a) I am not in a relationship<br><br>❍ b) I am currently in a relationship with one person<br>❍ c) I am currently in relationships with more than one person	If a) Have you been in a relationship in the past?<br><br>❍ Yes ❍ No<br><br>If yes, how long ago did your relationship end?<br>❍ Less than a week ago<br>❍ 1 to 4 weeks ago<br>❍ One month to 3 months ago<br>❍ More than 3 months ago
	(If b, c or yes to past relationship) Which best describes your relationship/s?<br><br>❍ Sexual relationship/s<br>❍ Non-sexual relationship/s
What gender is your partner (or ex-partner)?<br>(If you have more than one partner, please pick the one you are closest to or have been with the longest)<br><br>❍ Male<br>❍ Female<br>❍ Female to male transgender<br>❍ Male to female transgender<br>❍ Other (please state) ……………………………	

**Confidence about sex and relationships**	
When communicating about sex with a partner, how easy or difficult would it be for you to…….?	
1. Ask if they have ever had a sexually transmitted infection?	❍ Very difficult ❍ Difficult<br>❍ Easy ❍ Very easy ❍ Not applicable
2. Discuss contraception (birth control) (e.g. the pill)	❍ Very difficult ❍ Difficult<br>❍ Easy ❍ Very easy ❍ Not applicable
3. Discuss condom use?	❍ Very difficult ❍ Difficult<br>❍ Easy ❍ Very easy ❍ Not applicable
4. Refuse to have sex if they won't use a condom?	❍ Very difficult ❍ Difficult<br>❍ Easy ❍ Very easy ❍ Not applicable
5. Make the first move with sex	❍ Very difficult ❍ Difficult<br>❍ Easy ❍ Very easy ❍ Not applicable
6. Tell them that you like a specific sexual activity?	❍ Easy ❍ Very easy ❍ Not applicable
7. Tell them you do not want to have sex?	❍ Very difficult ❍ Difficult<br>❍ Easy ❍ Very easy ❍ Not applicable
8. Tell them if a certain sexual activity makes you uncomfortable?	❍ Very difficult ❍ Difficult<br>❍ Easy ❍ Very easy ❍ Not applicable
How confident are you that you could……	
1. Stop to use a condom in the heat of the moment?	❍ I definitely could ❍ I probably could<br>❍ I probably could not<br>❍ I definitely could not ❍ Not applicable
2. Put a condom on yourself or a partner without losing the erection?	❍ I definitely could ❍ I probably could<br>❍ I probably could not<br>❍ I definitely could not ❍ Not applicable
3. Suggest sex if you want it?	❍ I definitely could ❍ I probably could<br>❍ I probably could not<br>❍ I definitely could not ❍ Not applicable
4. Tell or show someone how they can give you sexual pleasure?	❍ I definitely could ❍ I probably could<br>❍ I probably could not<br>❍ I definitely could not ❍ Not applicable

Have you talked about these things with current (or most recent) partner/s? …..	
1. The kind of sex you like	❍ Yes ❍ No ❍ Not applicable
2. The kind of sex **a partner** likes	❍ Yes ❍ No ❍ Not applicable

**Sex and relationship problems**	
In the last 3 months, have you been….	
1. Humiliated or emotionally abused in other ways by a partner or ex-partner?	❍ Yes ❍ No ❍ Not sure

2. Afraid of a partner or ex-partner?<br><br>3. Forced to have any kind of sexual activity by a partner or ex-partner?<br><br>4. Kicked, hit, slapped or otherwise physically hurt by a partner or ex-partner?	❍ Yes ❍ No ❍ Not sure<br><br>❍ Yes ❍ No ❍ Not sure<br><br>❍ Yes ❍ No ❍ Not sure
In the last 3 months, **has a partner**..<br><br>1. Told you who you could see and where you could go<br><br>2. Pressurised you into any form of sexual activity?	 <br>❍ Yes ❍ No ❍ Not sure<br><br>❍ Yes ❍ No ❍ Not sure
In the last 3 months, have any of these been a problem for you?<br><br>1. Lacked interest in having sex<br>2. Lacked enjoyment in sex<br>3. Felt anxious during sex<br>4. Felt physical pain as a results of sex<br>5. Felt no excitement or arousal during sex<br>6. Did not come to a climax (experience an orgasm)<br>7. Came to a climax (experienced an orgasm) more quickly than you would like<br>8. (Women only) had trouble with an uncomfortably dry vagina<br>9. (Men only) had trouble getting or keeping an erection	 <br>❍ Yes ❍ No ❍ Not applicable<br>❍ Yes ❍ No ❍ Not applicable<br>❍ Yes ❍ No ❍ Not applicable<br>❍ Yes ❍ No ❍ Not applicable<br>❍ Yes ❍ No ❍ Not applicable<br>❍ Yes ❍ No ❍ Not applicable<br>❍ Yes ❍ No ❍ Not applicable<br>❍ Yes ❍ No ❍ Not applicable<br>❍ Yes ❍ No ❍ Not applicable
(If yes to any of these)<br><br>Have you avoided sex because of this?<br><br>❍ Yes ❍ No ❍ Not applicable	

In the last 3 months, how many times have you had sex you regretted?<br><br>None<br>1<br>2<br>3<br>4<br>5<br>6<br>7<br>8<br>9<br>10 or more	

**Satisfaction**	
Thinking about the past 3 months, how much do you agree or disagree with the following statements?<br><br>I feel satisfied with my relationship/s<br><br>I feel satisfied with my sex life<br><br>I feel distressed or worried about my sex life	❍ Strongly agree ❍ Agree<br>❍ Neither agree nor disagree ❍ Disagree<br>❍ Strongly disagree ❍ Not applicable

**The last time you had sex**	
When was the last time you had sex with someone (if ever)?<br><br>❍ Less than a week ago<br>❍ More than a week but less than a month ago<br>❍ 1-3 months ago<br>❍ More than 3 months ago<br>❍ Never	
What gender was the last person you had sex with?<br><br>❍ Male<br>❍ Female<br>❍ Transgender (male to female)<br>❍ Transgender (female to male)<br>❍ Other (please state) …………………	
Was the last person you had sex with…<br><br>❍ a. Somebody you've had sex with only once<br>❍ b. Somebody you've had sex with more than once	If b, how long have you been having sex with this person?<br><br>❍ Less than one week<br>❍ 1 week to 4 weeks<br>❍ 1 month to 6 months<br>❍ 7 months to 1 year<br>❍ More than 1 year
The last time you had sex…..<br><br>1. How safe and comfortable did you feel?<br><br>2. How much did you enjoy the physical feelings?<br><br>3. How emotionally close did you feel to the other person?<br><br>4. How much did you enjoy the pleasure you gave to the other person?	❍ Not at all ❍ Moderately ❍ Very safe and comfortable ❍ Not applicable<br><br>❍ Not at all ❍ Moderately ❍ A lot<br>❍ Not applicable<br><br>❍ Not at all ❍ Moderately ❍ Very close<br>❍ Not applicable<br><br>❍ Not at all ❍ Moderately ❍ A lot ❍ Not applicable
The last time you had sex, did you have vaginal sex, where the penis entered the vagina?<br><br>❍ Yes ❍ No ❍ Not applicable	(If yes) Was a condom used?<br><br>❍ Yes ❍ No ❍ Don't know ❍ Not applicable
	(If yes):<br><br>Was the condom used from start to finish of sex?

	❍ Yes ❍ No ❍ Don't know ❍ Not applicable
	Did the condom split or fall off?<br><br>❍ Yes ❍ No ❍ Don't know ❍ Not applicable
The last time you had sex, did you have anal sex, where the penis entered the anus?<br><br>❍ Yes ❍ No ❍ Not applicable	(If yes):<br><br>Was a condom used?<br><br>❍ Yes ❍ No ❍ Don't know ❍ Not applicable
	(If yes): Was the condom used from start to finish of sex?<br><br>❍ Yes ❍ No ❍ Don't know ❍ Not applicable
	Did the condom split or fall off?<br><br>❍ Yes ❍ No ❍ Don't know ❍ Not applicable
The last time you had sex, did you or your partner use any form of contraception (birth control)?<br><br>❍ a. None for me, don't know about partner<br>❍ b. None for either of us<br>❍ c. Yes (skip to list)<br>❍ d. Not applicable	(If c, yes) Please tick all contraceptives (birth control) that you used the last time you had sex:<br><br>❍ The Pill, contraceptive patch, or contraceptive vaginal ring<br>❍ Condoms (including female condoms)<br>❍ Emergency contraceptive pill (morning after pill)<br>❍ Injection<br>❍ Contraceptive implant<br>❍ Withdrawal<br>❍ Intrauterine device (coil/IUD/IUS)<br>❍ Diaphragm or cap or spermicide<br>❍ Natural family planning (safe period/rhythm method)<br>❍ Don't know name of the contraception<br>❍ Other (please state)…………………………
Are you or a partner trying to get pregnant at the moment?<br><br>❍ Yes ❍ No ❍ Not applicable	
Have you or a female partner been pregnant in the last 3 months?<br><br>❍ Yes ❍ No<br>❍ Don't know ❍ Not applicable	
(If yes) What happened with the pregnancy?<br><br>❍ Still pregnant<br>❍ Miscarriage or stillbirth<br>❍ An abortion<br>❍ A baby	

**More about sex**	

In the last 3 months, how many male partners have you had sex with?<br>0<br>1<br>2<br>3<br>4<br>5<br>6<br>7<br>8<br>9<br>10 or more	
In the last 3 months, how many female partners have you had sex with?<br>0<br>1<br>2<br>3<br>4<br>5<br>6<br>7<br>8<br>9<br>10 or more	
How many times in the last 3 months have you had vaginal sex without a condom?<br><br>❍ None<br>❍ Once<br>❍ Twice<br>❍ 3 times<br>❍ 4 times<br>❍ 5 times<br>❍ 6 times or more<br>❍ Not applicable	
How many times in the last 3 months have you had anal sex without a condom?<br><br>❍ None<br>❍ Once<br>❍ Twice<br>❍ 3 times<br>❍ 4 times<br>❍ 5 times<br>❍ 6 times or more<br>❍ Not applicable	
Which sexual health services have you used in the last 3 months (tick all that apply)<br><br>❍ None<br>❍ Condom pick-up	

❍ Contraception/birth control<br>❍ Emergency contraceptive pills<br>❍ Pregnancy test<br>❍ Discussion of abortion choices<br>❍ Check-up for sexually transmitted infections (e.g.<br>❍ Chlamydia, Gonorrhoea)<br>❍ Blood tests for sexually transmitted infections (e.g. ❍ HIV, syphilis or Hepatitis)<br>❍ Relationship counselling<br>❍ Sexual assault/abuse counselling<br>❍ Other sexual health services	
Adding up these visits, how many times did you use a sexual health service in the last 3 months?<br>1<br>2<br>3<br>4<br>5<br>6<br>7<br>8<br>9<br>10 or more	
Have you had Chlamydia in the last 3 months?<br><br>❍ Yes ❍ No ❍ Don't know	
Have you had antibiotic treatment for Chlamydia in the last 3 months?<br><br>❍ Yes ❍ No ❍ Don't know	
In the last 3 months**,** have you had any of the following (tick all that apply)?<br><br>❍ None<br>❍ Warts<br>❍ Herpes<br>❍ Gonorrhoea<br>❍ Pubic lice<br>❍ Trichomonas (TV)<br>❍ Syphilis<br>❍ HIV<br>❍ Hepatitis<br>❍ (WOMEN ONLY) Pelvic infection (PID)<br>❍ (WOMEN ONLY) Vaginal thrush (Candida, Yeast infection)<br>❍ Can't remember the name<br>❍ Other (please write in name)<br><br>………………………………………………	
In the last 3 months, how many times have you been too drunk or high to remember whether you had sex?<br><br>❍ Never<br>❍ Once<br>❍ Twice<br>❍ 3 times<br>❍ 4 times	

❍ 5 times<br>❍ 6 or more times	
How do you rate these sexual activities?<br>1. Sex by phone or online<br>2. Touching with clothes on<br>3. Touching with clothes off<br>4. Masturbating myself<br>5. Masturbating someone else<br>6. Being masturbated by someone else<br>7. Giving someone oral sex<br>8. Oral sex done to me	❍ Would like to try ❍Would **not** like to try<br>❍ Tried and would **not** do again<br>❍ Tried and would do again
1. Vaginal sex (penis-vagina)<br>2. Vaginal sex (with fingers or hand)<br>3. Vaginal sex (with sex toys)<br>4. Anal sex (rimming)<br>5. Anal sex (being rimmed)<br>6. Anal sex (penis-anus)<br>7. Anal sex (with sex toys)	❍ Would like to try ❍Would **not** like to try<br>❍ Tried and would **not** do again<br>❍ Tried and would do again

**In the future**	
In the near future, do you think you will……….	
1. Use a condom if you have vaginal sex with a new partner?	❍ Definitely not ❍Probably not ❍ Maybe- maybe not ❍ Probably ❍ Definitely ❍ Not applicable
2. Use a condom if you have anal sex with a new partner?	❍ Definitely not ❍ Probably not ❍ Maybe- maybe not ❍ Probably ❍ Definitely ❍ Not applicable
3. Have tests for sexually transmitted infections if you have a new partner?	❍ Definitely not ❍ Probably not ❍ Maybe- maybe not ❍ Probably ❍ Definitely ❍ Not applicable
4. Make sure that new partner/s have tests for sexually transmitted infections?	❍ Definitely not ❍ Probably not ❍ Maybe- maybe not ❍ Probably ❍ Definitely ❍ Not applicable
5. Make sure that you (or a partner) are using contraception (e.g. The pill)?	❍ Definitely not ❍ Probably not ❍ Maybe- maybe not ❍ Probably ❍ Definitely ❍ Not applicable
6. Discuss sexual enjoyment with partner(s)?	❍ Definitely not ❍ Probably not ❍ Maybe- maybe not ❍ Probably ❍ Definitely ❍ Not applicable

**True or false?**	
1. Whether or not I get a sexually transmitted infection is just luck.	❍ True<br>❍ False<br>❍ Unsure of answer
2. I would definitely know if I had Chlamydia, without needing a test	❍ True<br>❍ False<br>❍ Unsure of answer
3. You can easily tell who is likely to have Chlamydia	❍ True<br>❍ False<br>❍ Unsure of answer
True or false?<br><br>1. Baby oil or Vaseline is a good lubricant to use on a condom<br><br>2. With a condom on, the man should wait until the penis is soft before withdrawing after sex	 <br>❍ True ❍ False ❍ Don't know<br><br>❍ True ❍ False ❍ Don't know
True or false?<br><br>1. A woman's clitoris is right inside the vagina<br><br>2. Washing the vagina after penetrative sex (penis-vagina) will help to prevent pregnancy	 <br>❍ True ❍ False ❍ Don't know<br><br>❍ True ❍ False ❍ Don't know
Imagine that a 17 year old girl has had sex with her boyfriend about 10 times without condoms or contraception and didn't get pregnant<br><br>• This probably means she can't get pregnant<br><br>• This probably means that he can't get her pregnant	 <br><br><br>❍ True ❍ False ❍ Don't know<br><br>❍ True ❍ False ❍ Don't know

**Time to confess**	
For this research to be accurate, we need to be sure that only young people have completed the survey.<br><br>Are you really between 16 and 20 years old?<br><br>❍ Yes ❍ No	If no:<br><br>'Thank you for your interest in our survey, if you would like to know more about the research, please contact Ona McCarthy, o.mccarthy@ucl.ac.uk'
**About you**<br><br>These questions are to make sure that we've reached a mix of different young people in this survey.	
What is your date of birth?	

……..(Day)…………(Month) …………….(Year)	
Are you..<br><br>❍ At school<br>❍ At sixth form college<br>❍ At college or university<br>❍ In training<br>❍ Working<br>❍ Unemployed<br>❍ Long-term sick or disabled<br>❍ Other (please state) …………………….<br><br>(If you can choose more than one, please choose the option that best describes you)	
What is your cultural background?<br><br>❍ White British<br>❍ White Irish<br>❍ Other White<br>❍ Black British<br>❍ Black Caribbean<br>❍ Black African<br>❍ Other Black<br>❍ Asian British<br>❍ Indian<br>❍ Pakistani<br>❍ Bangladeshi<br>❍ Chinese<br>❍ Other Asian<br>❍ Mixed cultural background<br>❍ Other cultural background<br>❍ Prefer not to say<br><br>If other please state<br><br>……………………………………………………	
Who do you live with most of the time?<br>(tick all that apply):<br><br>❍ a. With parents or step parents<br>❍ b. With other relatives<br>❍ c. With friends<br>❍ d. With partner<br>❍ d. On your own<br>❍ e. In care or foster care<br>❍ g. With your children (or partner's children)	
Thinking about the people that you live with, are they…<br>(tick all that apply):<br><br>❍ At school, college or university<br>❍ In training<br>❍ Working<br>❍ Unemployed<br>❍ Long-term sick or disabled<br>❍ Not applicable	

❍ Other (please state) ……………………………………	

Is there anything else that you'd like to add?
Any comments about the answers that you gave, or about this online survey?

