# Supplementary material for: The Sexunzipped Trial: Young People’s Views of Participating in an Online Randomized Controlled Trial
Source: J Med Internet Res. 2013 Dec 12;15(12):e276. doi: 10.2196/jmir.2647 (PMC3868966; doi:10.2196/jmir.2647)
Supplement: Supplementary file 2 [file jmir_v15i12e276_app2.pdf]

Example Automated Emails for Sexunzipped Online Trial

Emailed prompt to use the websites at 6 weeks and 9 weeks from registration

Version 1 (No Chlamydia kit group)

Subject: Sexunzipped website

Curious about sex, relationships and sexual pleasure?

Visit sexunzipped.co.uk to find out more

Click on the 'Forgot your password' button on the homepage if you can't remember your password.

Just to remind you -

You can access the Sexunzipped website anytime until the study ends
We'll email you at the end of the study to ask you to fill in the Sexunzipped online survey.
We'll then send you your £10 shopping voucher through the post.

If you would like to withdraw from the study, please click the link below. You will not be contacted by us again. You will not be able to claim your £10 voucher if you withdraw before you have completed the second online survey.

Click here to withdraw from the study

Any questions? Please contact Ona McCarthy: o.mccarthy@ucl.ac.uk
Do not reply to this email

Email prompt to do the online questionnaire and Chlamydia sample at 13 weeks from registration

Version 2 (Chlamydia kit group)

Subject: Sexunzipped sexual health study

You signed up for the Sexunzipped sexual health study a few months ago.

It's now time to claim your £10 voucher.

How to claim your voucher:

Please go to www.sexunzipped.co.uk/3months and login to complete the Sexunzipped survey. We'd like to know if anything's changed for you since you joined the study three months ago.

You will also be getting a small pot through the post which is for a urine sample. When you post this back, our lab will test it for genital Chlamydia.

Once you complete the online survey and return the urine sample, we'll send you the £10 shopping voucher through the post.

Click on the 'Forgot your password' button on www.sexunzipped.co.uk if you can't remember your password.

If you would like to withdraw from the study, please click the link below. You will not be contacted by us again. You will not be able to claim your £10 voucher if you withdraw before you have completed the second online survey.

Click here to withdraw from the study

Any questions? Please contact Ona McCarthy: o.mccarthy@ucl.ac.uk

Do not reply to this email
